# Pairwise efficiency: a new mathematical approach to qPCR data analysis increases the precision of the calibration curve assay

**DOI:** 10.1186/s12859-019-2911-5

**Published:** 2019-05-30

**Authors:** Yulia Panina, Arno Germond, Brit G. David, Tomonobu M. Watanabe

**Affiliations:** 1 Laboratory for Comprehensive Bioimaging, RIKEN Center for Biosystems Dynamics Research (BDR), 6-2-3 Furuedai, Suita, Osaka, 565-0874 Japan; 2 Graduate School of Frontier Biosciences, Osaka University, 1-3 Yamadaoka, Suita, Osaka, 565-0871 Japan

**Keywords:** Quantitative PCR, Efficiency determination, Combinatorial treatment

## Abstract

**Background:**

The real-time quantitative polymerase chain reaction (qPCR) is routinely used for quantification of nucleic acids and is considered the gold standard in the field of relative nucleic acid measurements. The efficiency of the qPCR reaction is one of the most important parameters in data analysis in qPCR experiments. The Minimum Information for publication of Quantitative real-time PCR Experiments (MIQE) guidelines recommends the calibration curve as the method of choice for estimation of qPCR efficiency. The precision of this method has been reported to be between SD = 0.007 (three replicates) and SD = 0.022 (no replicates).

**Results:**

In this article, we present a novel approach to the analysis of qPCR data which has been obtained by running a dilution series. Unlike previously developed methods, our method, Pairwise Efficiency, involves a new formula that describes pairwise relationships between data points on separate amplification curves and thus enables extensive statistics. The comparison of Pairwise Efficiency with the calibration curve by Monte Carlo simulation shows the two-folds improvement in the precision of estimations of efficiency and gene expression ratios on the same dataset.

**Conclusions:**

The Pairwise Efficiency nearly doubles the precision in qPCR efficiency determinations compared to standard calibration curve method. This paper demonstrates that applications of combinatorial treatment of data provide the improvement of the determination.

**Electronic supplementary material:**

The online version of this article (10.1186/s12859-019-2911-5) contains supplementary material, which is available to authorized users.

## Background

Real-time qPCR is considered the most sensitive technique for nucleic acid quantification, and enables measurements on as few as several molecules of the target [1]. The advantage of this method over earlier methods of quantification, such as end-point PCR followed by gel visualization, is the ability to account for the efficiency of the PCR reaction by following it in real time and gathering fluorescence data after each amplification cycle [2–4]. The efficiency of the reaction is defined as the increase of product per cycle as a fraction of the amount present at the start of the cycle [5, 6]. In a classical model (for example, the one on which ΔΔCt method was based) it is assumed that the efficiency E of a qPCR reaction is stable and maximal before reaction saturation. The stability of E has been questioned numerous times [7–11], however, in our article we will be using the same assumptions as the classical model. Due to the exponential nature of PCR, the reaction efficiency can have dramatic effects on quantitative determinations. It has been estimated that an uncorrected 0.05 difference in amplification efficiency between a reference gene and a target gene can lead to false estimation of the target gene expression change of 432% [12].

The calibration curve method is widely considered the most precise method for qPCR efficiency estimation [13] and is required in the MIQE guidelines: “Calibration curves for each quantified target must be included with the submitted manuscript, slopes and y intercepts derived from these calibration curves must be included with the publication” [5]. The calibration curve is built by creating a serial dilution of known DNA concentration and plotting the quantification cycle (Cq) values on the y-axis against the logarithm of the sample concentrations on the x-axis. The efficiency (E) is then estimated from the slope of this curve using the classical formula E = 10–1/slope – 1; the estimation in this case is based on knowledge of the concentrations of all diluted samples. However, due to the insufficient precision of single dilution sets, it is often recommended to run at least three PCR reaction replicates for each sample to have three dilution sets for a single calibration curve. It has been shown that replicating a calibration curve three times by this approach increases the precision of efficiency estimation expressed as a confidence interval (CI) from 8.3 to 2.3% [13]. The downside of this approach is the increased workload and cost.

To overcome this problem of increased workload, several other methods have been developed to estimate qPCR efficiency from single curves and to improve qPCR precision in general, such as the PCR-Miner [14], LinRegPCR [15], sigmoidal fitting [16], and others. However, according to a recent analysis by Ruijter and colleagues, the majority of these alternative methods are very similar in principle as they are based on determining the same basic parameters (called Fq, Cq and E) and “all calculate a target quantity using an efficiency value and a Cq value” [6]. In addition, alternative methods that rely on different ways of approximating a single amplification curve have never yielded acceptable accuracy [17]. Thus, it remains to be seen whether a truly novel approach could improve the precision of qPCR efficiency and ratio estimations.

Here, we present a mathematical approach that improves the precision of qPCR efficiency estimation in the same number of samples that are required for calibration curve construction, thus reducing the necessary workload for qPCR. The aim of our method is to increase precision of qPCR efficiency estimation, as opposed to increase accuracy. Precision is defined as a measure of random error, in other words the error that arises due to random uncontrolled measurement variability, such as noise etc.; while accuracy is a measure of systematic error (e.g. an error that is “built into” experimental system due to, for example, systematic malfunction of equipment). Accuracy cannot be improved or determined by any statistical manipulations of the data, and correction of accuracy requires a comparison of the results to an already known standard. Since such standards (e.g. standard sample of ideal efficiency for the actin gene) do not exist in biology yet, the aim of our work was to decrease the magnitude of random, measurement-related error. In other words, since it is currently impossible to know the “true” amplification efficiency of any gene due to lack of internationally recognized standard samples, our statistical method concerns precision only, as do all other previously developed methods.

Our approach relies on pairwise relationships between fluorescence (not Cq) readings on several amplification curves of a dilution set. We employ the following strategy to increase the precision of determinations. First, we introduce a new formula for efficiency (E) estimation based on the relationship between data points on each of the amplification curves from the dilution set. This approach allows us to increase the number of determined unique E values to hundreds. Second, using this array of unique E values, we perform standard statistical analyzes such as the analysis of value distribution, and the exclusion of outlier values. The statistical analysis becomes possible precisely due to the fact that we generate hundreds of data points, as any statistics quality depends on the number of unique values in any given set.

Our results show that the application of Pairwise Efficiency makes it possible to nearly double the precision in qPCR efficiency determinations without increasing the pipetting workload and minimizing cost. In addition, we demonstrate a 2.3-fold improvement in precision of the estimation of gene expression ratios. This constitutes a conceptual advance in the field of qPCR and allows for further development of ideas in this direction. Moreover, these advancements have important practical implications for the use of qPCR.

## Methods

### DNA sample

Mouse embryonic stem cell line E14Tg2a was purchased from RIKEN Cell bank, JP (AES0135) and was maintained as previously described [18]. Total RNA was extracted using RNeasy kit (Cat# 74106, Qiagen, Japan) following the manufacturer’s instructions. Genomic DNA digestion was performed on-column according to said instructions. RNA concentration and absorbance ratios (A_260/280_ and A_260/230_) were checked with a spectrophotometer Nanodrop 2000 Spectrophotometer (NanoDrop Technologies, Japan). To produce cDNA for qPCR analysis, 300 ng of total RNA were reverse-transcribed with an Omniscript RT Kit (Cat# 205111, Qiagen) in a total volume of 20 μl. The resulting DNA concentration was assessed by spectrophotometric analysis and diluted to 100 ng/μl.

### Quantitative real-time PCR setup and reagents

qPCR was performed using a CFX96 Connect apparatus (Bio-Rad, Japan). Hard-Shell® 96-Well PCR Plates (Cat # HSP 9601, Bio-Rad) sealed with optically clear adhesive seals (Microseal® ‘B’ seal, Cat # MSB1001, Bio-Rad) were used in all experiments. The thermocycler program consisted of an initial hot start cycle at 95 °C for 3 min, followed by 33 cycles at 95 °C for 10 s and 59 °C for 30 s. Mouse actin beta (Actb) was amplified using the following primers: F-5′-AACCCTAAGGCCAACCGTGAA-3′, R-5′-ATGGCGTGAGGGAGAGCATA-3′ (with estimated product length 194 bp). The primers were used at a concentration of 300 nM. SYBR Green-based PCR supermix (Bio-Rad) was used for all reactions according to manufacturer’s instructions. Each reaction was performed in a final volume of 8 μL. To confirm product specificity, a melting curve analysis was performed after each amplification, and agarose gel analysis was performed to ensure the amplification of the right product (Additional file 1: Figure S1).

### Experiment design and PCR dataset generation

For the assessment of precision of our method and comparison with the classical calibration curve method, we produced 16 replicas of a 6-step dilution series. We provide the detailed pipetting layout in Additional file 1: Figure S2. Two datasets were generated from this experiment and processed using Bio-Rad CFX Manager 2.0 (2.0.885.0923). Additional file 2: Dataset 1 consists of relative fluorescence data obtained from the aforementioned experiment: 6 serial dilution wells × 16 replicas = 96 wells. Fluorescence data in Additional file 2: Dataset 1 are expressed as RFU (Relative Fluorescence Units) which is a term specific to Bio-Rad software. It is important to note that, since our goal was to improve the accuracy of the classical calibration curve, all RFU values were taken as already processed by Bio-Rad software with the same settings that were applied to the generation of Cq values, as follows: Baseline Setting set to Baseline Subtracted Curve Fit, Cq Determination Mode set to Single Threshold. Additional file 3: Dataset 2 contains automatically generated Cq values corresponding to Additional file 2: Dataset 1. The threshold was automatically set at 31.07 by the Bio-Rad software.

### Determination of the exponential region

The most suitable bounds of the exponential region of the respective amplification curves were determined experimentally. Prior to the experimental estimation, we conducted an initial estimation using the “first outlier” method and the First Derivative Maximum (FDM) approach [9, 19]. The initial estimation was done solely in order to provide a general range for experimental testing. The results of the formula of “first outlier” detection [19] application to the first calibration curve replica (wells A1 through A6) are provided in Additional file 1: Table S1. In agreement with these data, the tentative lower boundary of the exponential region was set at 10–40 RFU. The FDM values for the first calibration curve replica can be found in Additional file 1: Table S2. As expected, the values differ for samples with different initial DNA concentration, and are in the range of 18–25 cycles for FDM values. Additional file 1: Figure S3a shows the FDM values for the whole Additional file 2: Dataset 1 plotted against cycle numbers. The earliest FDM was encountered at cycle 18 in the most concentrated sample. The latest FDM of the dataset came at cycle 25. As shown in Additional file 1: Figure S3b, the RFU values for cycles corresponding to calculated FDMs fall in the range of 120–230 RFU. Thus, in accordance with these data, the tentative initial estimation of the upper boundary of the exponential region to use in the experimental test was set between 120 and 230 RFU.

### Determination of the best-performing boundaries in the exponential region

As shown in the previous section, the exponential region of each curve in a dilution set starts at a different cycle. Thus, it is necessary to experimentally determine the most suitable upper and lower boundaries of the exponential region for all curves taken together. To determine the most suitable boundaries, we experimentally tested at what fluorescence range (i.e. what portion of each of the amplification curves) the application of our method produces results with the highest precision. For this estimation we applied a “Monte Carlo” approach that was previously described by Svec et.al. for the evaluation of precision of the calibration curve method [13]. The lower boundary was tested at the range of 10 RFU - 80 RFU, and the higher boundary was tested at the range of 120 RFU - 230 RFU. Exact boundaries tested can be found in Additional file 1: Table S5 (altogether 10 combinations of boundaries, which we wanted to compare for precision performance). Using fluorescence RFU readings from Additional file 2: Dataset 1 that contained 16 technical replicas of a 6-step dilution set, we randomly drew 100 different “samplings” (or sub-populations) consisting of three six-sets, from the general population of 16 (Additional file 1: Figure S4), and calculated the precision for each combination of varying boundaries expressed as standard deviation (SD). The results of this operation are displayed in Additional file 1: Table S5 and visualized in Additional file 1: Figure S4. The best results were obtained at the lower portion of the curve (40–120 RFU). The variation in the SD value did not exceed 0.001 for the lower portion (40–120 RFU, 40–150 RFU, 20–150 RFU). To include as many values as possible in our case, we decided to use 20–180 RFU boundaries, which produce smallest SD while including approximately 4 fluorescence data points.

### Baseline treatment

Since the goal of our analysis was to directly improve the precision of the classical calibration curve method, the same software settings were applied to fluorescence data as to the generation of Cq values. The Bio-Rad software was set to Baseline Subtracted Curve Fit, and the baseline was subtracted automatically by the software producing Relative Fluorescence Unit values. This Bio-Rad subtraction method is based on either adding a constant value, or a linearly growing value to the raw fluorescence and thus does not eliminate the noise inherent to any qPCR instrument as an electric device.

### Evaluation of the noise influence

To determine the properties of noise and the scale of noise influence, we examined the fluorescence readings in the beginning cycles of the Additional file 2: Dataset 1. As shown in Additional file 1: Figure S6a, the fluorescence readings in the beginning cycles (up to cycle 13–18, depending on the starting concentration) were distributed close to 0, with inclusion of negative readings. The minimal value of the whole dataset was − 9.44 RFU. To demonstrate the noise distribution, we show three histograms which contain fluorescence readings from the following cycles: 1) Cycles 1 through 5; 2) Cycles 1 through 10; and 3) Cycles 5 through 10. The data were taken from Additional file 2: Dataset 1 and two more 96-well plates replicating serial dilutions, with the Actb gene as target (raw data of these two plates are available on request). The total number of data points resulted in 2880 fluorescence readings (first 10 cycles from 96 wells in 3 plates). The result is shown in Additional file 1: Figure S6b. The noise in the beginning cycles appeared to have a nearly normal distribution with a non-zero peak. The positions of the peaks and the distribution did not change depending on the number of included cycles, which indicated that there was no detectable signal at this stage - because the increasing signal would have produced a shift to the right in the noise distribution if it existed. Thus, we concluded that the initial fluorescence readings in our system contain noise, and the noise has the approximate range of − 10 RFU to 10 RFU. To ensure that all data points that we would take for analysis contain the non-noise signal, we concluded that the lower boundary should not be lower than 10 RFU which is in accordance with the boundary set by the ‘first outlier’ (see Determination of the exponential region).

### Data processing

The data processing was carried out in Microsoft Excel and R. All excel files are available in Additional files 2 and 3.

## Results

### Assessment of the detectability of stable amplification efficiency in the exponential phase

The goal of our analysis was to increase the accuracy of measuring the mean amplification efficiency that is normally determined by the classical calibration curve method [5] as opposed to cycle-to-cycle efficiency described in other models. According to the mainstream view, any PCR reaction proceeds with stable efficiency until end-stage reagent depletion and the accumulation of reaction products cause a steep decline in the efficiency, and the reaction gradually slows down [20, 21]. The calibration curve method aims at measuring the stable efficiency of the reaction before the saturation occurs, and this maximal efficiency is assumed to be identical across all dilution samples. However, it has been argued that the sensitivity of some qPCR machines does not allow detection of a weak fluorescent signal in the exponential phase of the PCR reaction, where the efficiency is still stable, and the signal first appears when the efficiency is already declining [7, 9, 22]. It has also been pointed out that the analyzes based on stable efficiency should be conducted strictly at the region before efficiency decline, if such a region is detectable.

To determine if our system allows to detect the theoretical stable efficiency, we analyzed the fluorescence readings data from Additional file 2: Dataset 1 (see Materials and Methods for description) using the following formula for the calculation of efficiency E.

1 $$ E={2}^{\frac{{\mathit{\log}}_2{F}_i-{\mathit{\log}}_2{F}_0}{i}}-1 $$

, where *i* is the cycle number for a particular fluorescence reading F, and F_0_ is the initial fluorescence value of the sample. The logarithms, base 2, are used because the series contains 2-fold dilution sets.

**Fig. 1:**
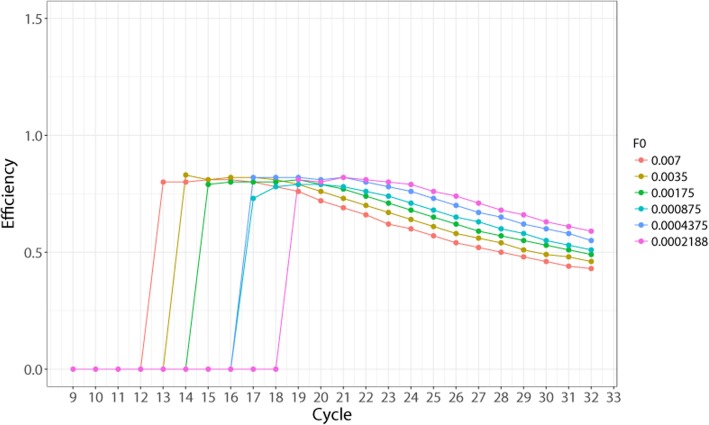
A graphical representation of the efficiency (E) values across all cycles taken from a 6-step dilution set. Efficiency is calculated using the formula $E={2}^{\frac{{\mathit{\log}}_2{F}_i-{\mathit{\log}}_2{F}_0}{i}}-1$. The F_i_ and i values for calculation are taken directly from Additional file 2: Dataset 1, wells A1 through A6. Since F_0_ value is unknown, it was selected from the range of theoretically possible F_0_ values (covering 0.007–0.0002) and used in the formula

The formula (1) cannot be used directly for E calculation because the fluorescence level of the starting material F0 is unknown. The purpose of the analysis described below was to confirm the detectability of the stable exponential E region with varying F0 values. To obtain initial approximation of F0 value to test with formula (1), we used E values calculated using calibration curve method (Additional file 1: Table S3). Knowing the efficiency of the reaction (around 80%) allowed us to produce initial F0 estimations by the standard formula. The resulting F0 values were in the range of 0.007 to 0.0002. We then substituted these F0 values in the formula (1) and analyzed the resulting E values at each cycle of the reaction (Fig. 1). As shown in the figure, we found that in the first cycles where non-background signal is detected by the machine, E displays a relatively constant pattern (SD = 0.01), while in the later cycles it starts to decline steadily (Additional file 1: Table S4). The initial region with the small standard deviation lasted from cycle 13 until cycle 17 for the most concentrated sample. Varying the F0 value did not affect the detection of this region of relatively constant E, as other curves also produced a similar pattern with small variation of E in the initial 3–5 cycles where the signal was already detected, and a steady decline after that.

According to these data, our experimental system allowed the detection of approximately 4 fluorescence values from the exponential phase of amplification where the variation of efficiency does not exceed ±0.01. This result overall shows that the theoretical stable efficiency is detectable and can be quantified.

### Amplification efficiency estimation

Next, we approached the question of how to reduce the uncertainty in the estimation of E given that only 5 or fewer fluorescence data points on each curve belong to the E stability region.

**Fig. 2:**
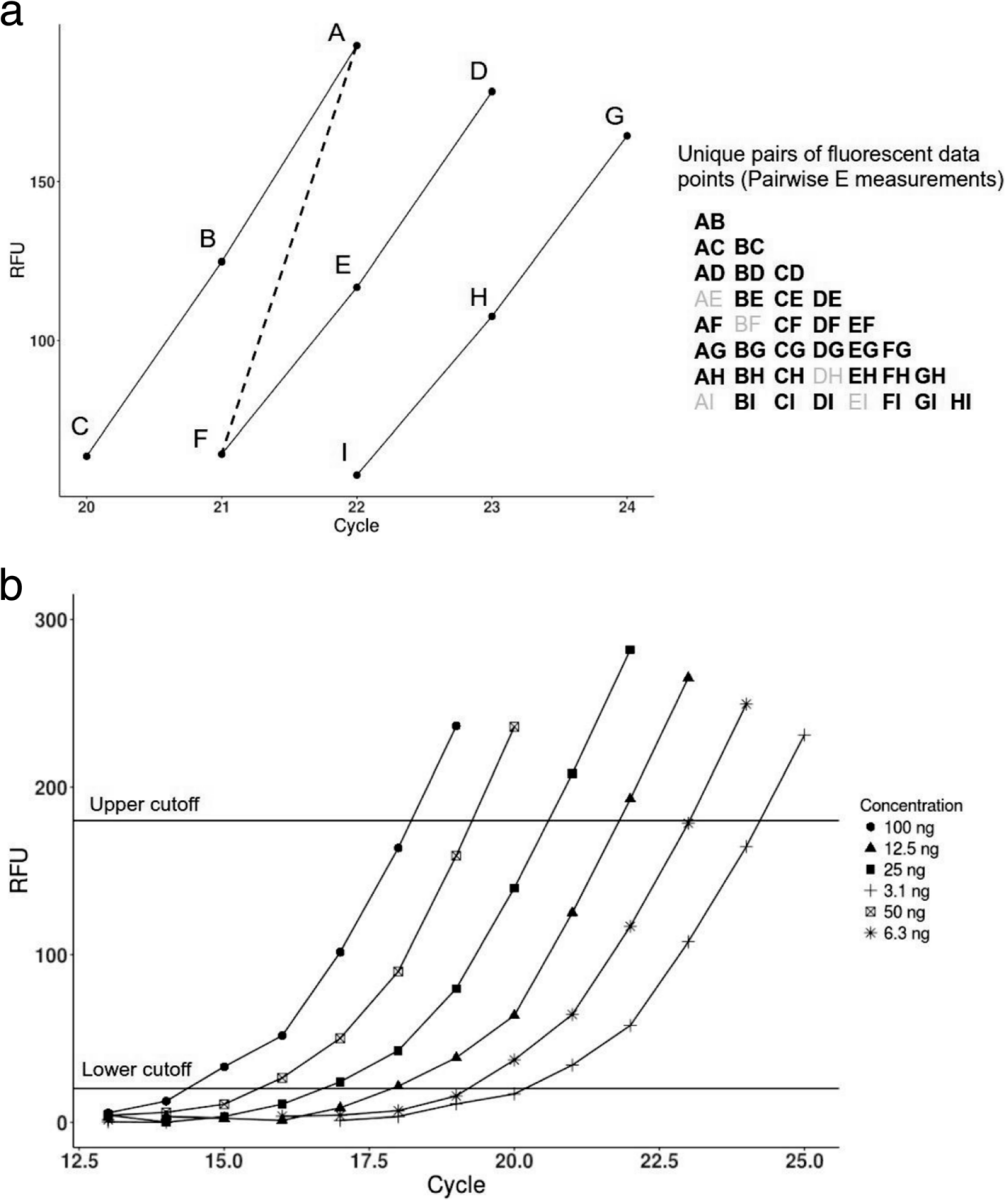
Graphical representation of the principle of Pairwise Efficiency method and its application to six dilution curves. (a) A graphical illustration of the Pairwise Efficiency method. Small portions of three amplification curves, with three fluorescence data points on each, are shown. Dashed line connects point A to point F on separate curves, and represents a single, unique pairwise E determination (pair AF). All possible pairs, each one representing a unique pairwise E value, are shown on the right. Since some of the values occur on the same cycle (for example, AE, BF), such values are excluded from the determinations, and are denoted in gray. (b) The amplification curves from the wells C1 through C6 are shown (RFU data taken from Additional file 2: Dataset 1). Different shapes (circles, squares, triangles etc.) represent fluorescence readings taken by the machine after each PCR cycle. Horizontal lines denote the region of amplification curves from which the fluorescence data points were taken for analysis. Upper cutoff was set at 180 RFU, and lower cutoff was set at 20 RFU. In this experiment, the total of 24 fluorescence data points fall inside the denoted region, and unique pairs formed by these 24 points, excluding repetitive values occurring on the same cycle, are taken for analysis

For this purpose we introduced a new formula (4) for E estimation from a dilution set. This formula describes the relationship between 2 individual fluorescence readings in any given dilution set. The fluorescence readings are represented by data points on 6 amplification curves, in the case of one 6-step serial dilution experiment (Fig. 2b). The E estimation in our case is based on a relationship between a pair of actual fluorescence readings, as opposed to the slope of the calibration curve, which is based on cycle fraction values (Cq).

When devising our formula, we used the same basic assumptions that the calibration curve method uses [6, 23] when calculating the mean efficiency on a calibration curve, namely:

1. The kinetics of a PCR reaction with a given DNA-primer set are the same irrespective of the initial template concentration.
2. The kinetics of the PCR reaction are assumed to be classical (described by the classical formula F=F0 × (1 + E)^i^)
3. The mean efficiency is maximal and constant before the reaction saturation.
4. Fluorescence readings and double-stranded DNA concentration are linearly related to each other, and the increase in fluorescence is directly proportional to the increase in target concentration.

Given these assumptions, any one fluorescence reading F on any one of the amplification curves in the dilution set can be described by the following equations:

2 $$ {F}_i=\frac{F_0}{2^{D1}}\times {\left(1+E\right)}^i $$

3 $$ {F}_j=\frac{F_0}{2^{D2}}\times {\left(1+E\right)}^j $$

, where *i* and *j* are cycle numbers for a particular fluorescence reading, F_i_ and F_j_ are the fluorescence readings in cycle *i* or cycle *j*, F_0_ is the initial fluorescence of the undiluted sample, D1 and D2 are dilution factors for curve 1 and curve 2 (if the pair of data points are on the same curve, then D1 = D2), and E is the amplification efficiency for the qPCR reaction for the given DNA-primer set. The dilution factor D is defined as the logarithm of the fold-dilution, compared to the undiluted sample whose logarithm of the fold-dilution, by definition, is 0. Since we applied twofold dilutions for mathematical clarity, D values in our case were integers from 0 to 5. In the case of tenfold dilutions, the corresponding ‘2’ values in the formulae will become 10, and the dilution factors will remain unchanged.

The eqs. 2 and 3 allow us to calculate the efficiency E for a given pair of fluorescence readings, such as:

4 $$ {E}_{i,j}={2}^{\frac{\left({\mathit{\log}}_2\left({F}_j\right)-{\mathit{\log}}_2\left({F}_i\right)+\left(D2-D1\right)\right)}{\left(j-i\right)}}-1 $$

Thus, while the estimation of efficiency across a dilution set by the calibration curve method is based on a single curve and produces a single E value, our new method, Pairwise Efficiency, calculates an array of E values based on all possible pair combinations from this dilution set, producing about 50–400 individual pairwise E determinations (depending on the number of fluorescence readings included in the exponential region taken for analysis), and then estimates the average efficiency from this array of E determinations.

### Statistical analysis of the array of resulting efficiency (E) values

**Fig. 3:**
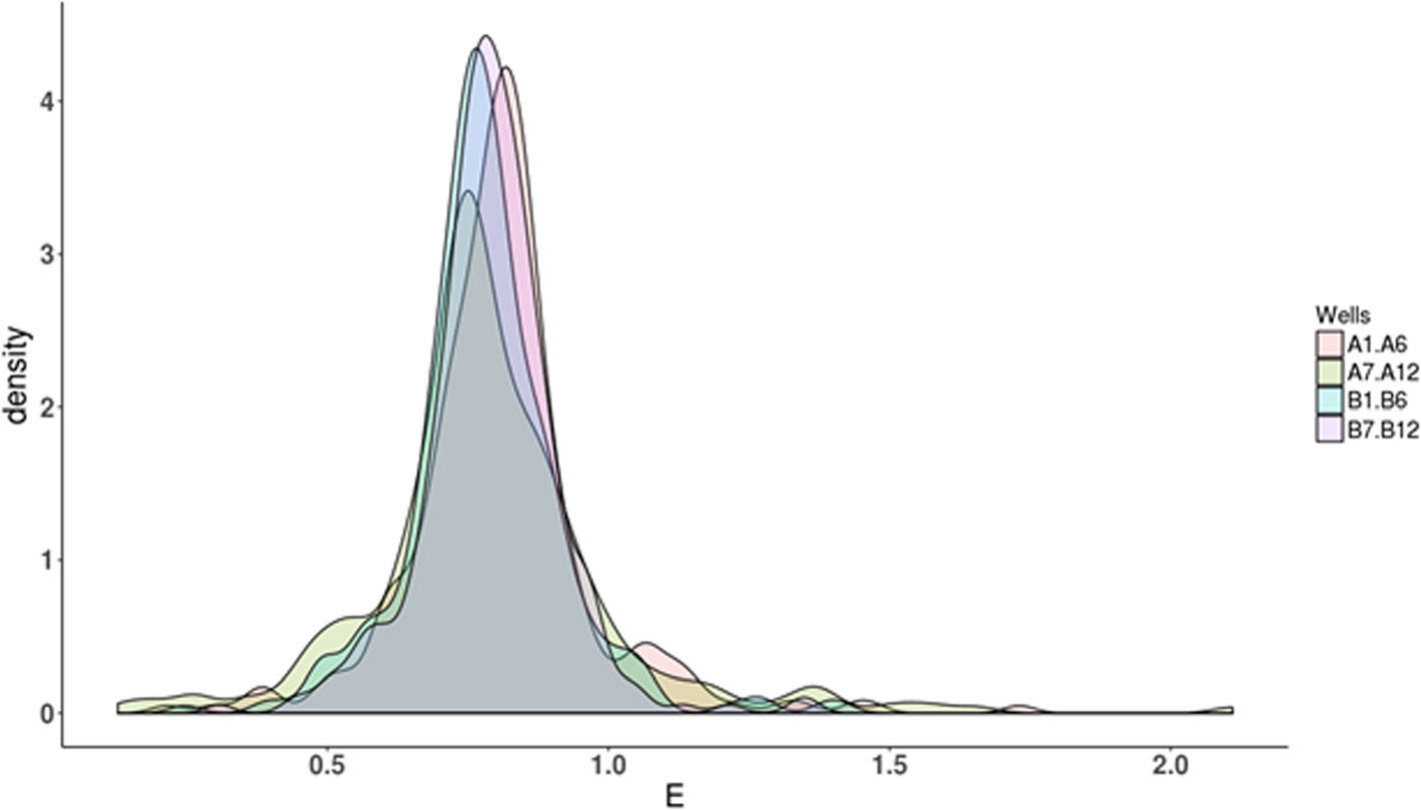
A graphical representation of the distribution of pairwise E values for the wells A1-A12 and B1-B12. The distribution of pairwise E values is leptokurtic in all sets, and has a sharp appearance, indicating that the values are closer to mathematical expectation, and precision is higher than would be expected in the case of normal distribution. In addition, the distributions are skewed and possess larger tail areas, indicating significant deviation from normality

To further improve the precision of estimation of Pairwise Efficiency, we considered methods to remove outliers, which aims at excluding unreasonable values that occur due to random measurement errors, as to increase the precision of determinations. First, we analyzed the distribution of pairwise E values for normality in each group of pairwise E determinations. This analysis is necessary in order to decide which kind of method to use for outlier exclusion (parametric, such as three sigma rule, vs. non-parametric). To assess the distribution normality in a mathematically objective way, we used standard tools, namely, skew, kurtosis, and chi-square test. As shown in Table 1, the majority of skewness values significantly deviated from 0, confirming distribution asymmetry. In addition, all kurtosis values were positive, indicating that calculated pairwise E determinations from these dilution sets had leptokurtic distribution (Fig. 3).

**Table 1:** Estimation of distribution normality

Dilution set (wells)	Skew	Kurtosis	Total data points
A1–6	1.064	7.357	237
B1–6	0.615	4.085	237
C1–6	0.221	3.556	244
D1–6	1.051	6.305	241
E1–6	0.473	5.524	240
F1–6	1.88	6.769	222
G1–6	2.012	10.079	220
H1–6	1.379	12.177	223
A7–12	−0.337	2.16	220
B7–12	0.098	4.508	217
C7–12	0.215	2.838	259
D7–12	0.739	2.514	241
E7–12	0.563	3.555	223
F7–12	−0.034	3.843	206
G7–12	1.429	7.023	198
H7–12	−0.148	5.319	240

Pairwise E values of 16 dilution sets were analyzed for skewness and kurtosis. Skewness values that deviate from 0 indicate asymmetry of the distribution, making it a non-normal distribution. Positive kurtosis values also imply deviation from normal distribution and indicate that the distribution is sharp (more values are close to mathematical expectation, and precision is higher than would be expected in the case of normal distribution). The right column contains the numbers of individual pairwise E determinations for each dilution set that were taken for this analysis

Next, we used the Pearson’s chi-squared test to test the goodness of fit of the frequency distribution of calculated pairwise E values. When analyzing 16 curves, we have an average standard deviation value of 0.116 over 16 replicas. Therefore, we used the interval length of 0.05, as required by chi-square test. The details of our chi square test calculations are shown in Additional file 1: Table S6. The application of chi-square test is considered valid if there are at least 50 values analyzed for distribution (which is the case of Pairwise Efficiency), and no more than 20% of the values have expected frequencies below 5. The values whose frequency is less than 5 are considered statistically unreliable and are designated as outliers. An analysis by the Chi-square test showed that our distributions significantly deviated from normal (Additional file 1: Table S6). Thus, parametric tools designed for normally distributed values, such as quartile ranges or sigma rules, could not be applied in this case. Instead, when the distributions do not follow a fixed set of parameters (e.g. are not normal), non-parametric statistical tools are used; however, the selection of specific tool is left to the researcher and is decided case-by-case. Since Pearson’s chi-square test is a universal tool that can be applied to any kind of distribution (both parameterized and non-parametrized), we chose to use the criteria of this test to exclude outlier E values in our case. As mentioned above, according to the principles of the Pearson’s chi-square test, the values whose frequency is less than 5 are considered statistically unreliable. Based on this criterion, the pairwise E determinations with frequency less than 5 were considered outliers and were excluded from the calculation of the E value of one dilution set.

Thus, for example, the dilution set in wells A1 through A6 had 167 individual pairwise E determinations, skewness = 1.06 and kurtosis = 7.36. The frequency of E values below 5 was first encountered at E = 0.6 (60% efficiency) on the lower end, and at E = 1.05 (105% efficiency) on the higher end (Additional file 1: Table S7). Based on Chi-square criterion, all pairwise E determinations that exceeded 105% and did not reach 65% were excluded from the calculation of average E for this dilution set. E value for wells A1 through A6 prior to outlier analysis was E = 0.79, and after the removal of outliers became E = 0.816. Other E values for the remaining 15 sets were processed on the basis of the same algorithm.

### Comparison of the performance of pairwise efficiency method vs. the calibration curve-based E estimation

Next, we set out to compare the *precision* of our method to the classical calibration curve method. Since precision is defined as a measure of random error, it can be investigated by the same Monte Carlo approach that was used for comparison of different boundaries described in Materials and Methods. In this case, we did not vary the boundaries (because the purpose was not to compare the precision of varying boundaries) but varied the approach instead: E calculated by classical calibration curve method vs. E calculated by Pairwise Efficiency method. Again, to produce precision estimation, we randomly took 100 “samplings” (or sub-populations) consisting of three replicates of 6-times dilution sets (Additional file 1: Figure S4). Thus, one “sampling” would produce three separate E values because one 6-times dilution set yields one E estimation (MIQE guidelines). These three E values in a “sampling” were averaged, as required by MIQE. Then, this procedure was repeated 100 times to produce 100 “samplings”, and SD was found for all of them. The SD was found to be 0.019. Next, we applied the same approach to the corresponding RFU values (not Cq this time) on exactly same qPCR plate and exactly same samples, with only difference that E was calculated by Pairwise Efficiency. The results are shown in Table 2. Pairwise Efficiency produced an increase in the precision of E estimation from 0.010 to 0.019, thus nearly two-fold. While the average E values were found to be 80% in both methods, Pairwise Efficiency produced a smaller standard deviation and a smaller difference between maximal and minimal E values. The dispersion of E values obtained by Pairwise Efficiency method, expressed as Max E - Min E, did not exceed 0.045, as opposed to 0.072 obtained by the calibration curve method. This means that the magnitude of random error in the E estimation was approximately two times lower in the case of Pairwise Efficiency compared to the calibration curve method.

**Table 2:** Comparison of the calibration curve method with the Pairwise Efficiency method

Approach	SD	Max E	Min E	Max-Min difference	Average E
Calibration curve	0.019	0.83	0.76	0.072	0.80
Pairwise Efficiency	0.010	0.82	0.78	0.047	0.80

Standard deviations (SD) obtained from the Monte Carlo test, maximal and minimal efficiency values, the range between maximal and minimal values, and the average efficiencies are shown. While the average E value was the same for both methods (E = 0.80), the precision of E estimation obtained by the Pairwise Efficiency method, expressed as standard deviation (SD), was nearly two times higher, and the dispersion, expressed as the difference between maximal and minimal calculated E values, was 1.6 times smaller

Next, we investigated whether this increased precision in the efficiency estimation would translate into increased precision of gene expression ratio measurements. To do that, we calculated the magnitude of possible error for the calibration curve method and for the Pairwise Efficiency method, using the same assumptions as described in Materials and Methods. For the calculation of expression ratios in the case of calibration curve, we used the equations described by M. Pfaffl [24]. The mathematical model presented in his publication is, in principle, equivalent to the model previously designed by Roche Diagnostics and takes into account the efficiency of both target and reference genes. The formula presented by Pfaffl has the following appearance:

$$ ratio={E}_{target}^{\Delta  Ct} $$

, where ΔCt is the difference between Ct of the sample and Ct of control at the same threshold. Since our dataset of 16 dilution replicas contained exactly the same amount of target gene (Actb) in wells with the same concentration, theoretically the calculated ratio between these wells should be 1. Thus, we could evaluate the magnitude of error in the determination of the ratio by measuring maximal difference between each one of these 16 replicas. In this case, the error would be maximal when the efficiency value is maximal.

First, we determined which one of the 16 dilution sets gives the highest efficiency value. The analysis using the calibration curve method showed that wells D1 through D6 produced the highest efficiency (E = 0.882). Next, using this efficiency, we applied the aforementioned formula for the undiluted samples, considering the Ct _sample_ the highest Ct from all 16 replicas, and Ct _control_ the lowest of all. This resulted in a ratio = 1.606. Thus, the maximal possible error in the estimation of gene expression ratio when using the calibration curve method can reach up to 60%. Similarly, we used the maximal efficiency calculated by Pairwise Efficiency method to estimate the magnitude of error on Additional file 2: Dataset 1 with 16 replicas. The maximal efficiency value was obtained in the same wells (D1 through D6) as for the calibration curve, which underscores robustness of both methods for E estimation. Using this maximal efficiency value, we estimated F_0_ in all wells using our modified formula (2):

$$ {F}_0=\frac{F_i}{{\left(1+E\right)}^i} $$

, based on actual fluorescence values. The estimation of F0 in our Pairwise Efficiency method in this case was analogous to the calibration curve method, while the way we estimate efficiency differed. We obtained the following result: Max F = 0.00435436, Min F = 0.00345735. Then we calculated the difference between maximal F_0_ and minimal F_0_ which yielded a ratio = 1.26. Thus, the magnitude of possible error in ratio estimation using Pairwise Efficiency method amounts to 26%, which amounts to an improvement of about 2.3 fold in the precision of gene expression ratio estimation compared to the calibration curve method.

Then, we compared the performance of Pairwise Efficiency vs. calibration curve in terms of accuracy. Since accuracy is a measure of systematic error, it can only be determined by comparing the result to a known standard. International biological standards for RT-qPCR do not exist. Thus, it is only possible to determine accuracy indirectly, for example, by comparing the resulting determinations to a chosen standard of another known value (such as dilution proportions which are known etc.) For this comparison, we calculated the error in determination of the dilution ratio because in our case the dilution ratios were known (Table 3).

**Table 3:** Comparison of the accuracy between Pairwise Efficiency and the standard calibration curve method based on a chosen standard

Wells	Conc.	Efficiency	F0	Ratio (PE)	Error (%)	Ratio (Ct)	Error (%)
A1-A6	100 ng	0.73130	0.00800	1	N/A	1	N/A
A7-A12	100 ng	0.76200	0.00780				
B1-B6	100 ng	0.77170	0.00660				
B7-B12	100 ng	0.77230	0.00710				
C1-C6	50 ng	0.83530	0.00280	2.513	20%	2.47	19%
C7-C12	50 ng	0.79550	0.00290				
D1-D6	50 ng	0.81870	0.00290				
D7-D12	50 ng	0.82390	0.00300				
E1-E6	12 ng	0.75780	0.00060	8.519	6%	12.73	37%
E7-E12	12 ng	0.68420	0.00110				
F1-F6	12 ng	0.72470	0.00090				
F7-F12	12 ng	0.70420	0.00100				
G1-G6	3 ng	0.76180	0.00020	35.455	10%	57.41	44%
G7-G12	3 ng	0.66870	0.00020				
H1-H6	3 ng	0.72810	0.00020				
H7-H12	3 ng	0.66640	0.00020	Aver error:	12%		33%

The efficiency of amplification of Actin beta was determined using Pairwise Efficiency or the standard calibration curve method (for standard method E values see Additional file 1: Table S3). The known dilution ratio (differences between DNA template concentrations) were used as a reference. 100 ng was taken as 1, and thus all diluted samples should have yielded the following values: 2 (for 50 ng), 8 (for 12 ng) and 32 (for 3 ng). The error values in determining the correct ratios were lower than those calculated by standard method. The average error for Pairwise Efficiency was equal to 12%, while the average error for standard method was equal to 33%

This result demonstrates that Pairwise Efficiency can produce more accurate estimations of template quantity than the calibration curve approach (described in MIQE) in the same experiment with the same number of pipetted wells.

Finally, to confirm the universality of Pairwise Efficiency method, we have applied it to different baseline settings (in our case, “Baseline Subtracted” and “Baseline Subtracted Curve Fit”), as well as to 10-fold dilution series. The results can be found in Supplementary Information (Additional file 1: Tables S8, S9, S10).

## Discussion

Quantitative PCR is an affordable and widely used technique for nucleic acid quantification. However, despite its popularity, this method has yet to gain full acceptance in the research community due to limitations in its ability to provide precise determinations, which may lead to low reproducibility. Multiple methods for qPCR data analysis have been developed throughout its history, yet the vast majority of these relies on Cq values, as well as a calibration curve or curve fitting for efficiency estimation and subsequent data analysis. Moreover, such previous methods do not achieve sufficient improvement in precision of estimations of efficiency or gene expression ratio. Thus, new approaches are needed to overcome the limitations of existing methodologies. In this report, we introduce a new approach to qPCR data analysis, Pairwise Efficiency, which consists of three elements. First, it introduces a formula describing the relationship between two fluorescence readings on amplification curves and does not rely on Cq values or a calibration curve for the estimation of reaction efficiency. Second, it estimates the boundaries of the exponential region for a group of amplification curves in order to determine reliable data boundaries. And third, it eliminates outliers during the process of calculating E values, as opposed to at the end.

It should be noted that the PCR efficiency determined from a dilution series calculates an ‘average’ efficiency with an equation that includes the intended dilution of the samples (Eq. 4). Therefore, an error in the actual dilution of the samples leads to a systematic error in the measured fluorescence values and thus to a bias in the observed PCR efficiency values. Indeed, when we analyzed the PCR efficiency by standard method and Pairwise Efficiency method in case of 10-times dilutions, the efficiency values themselves were slightly different (Additional file 1: Table S10). The difference observed between the efficiency values in the 2-times and 10-times diluted series may be due to such a systematic error in pipetting the dilution series.

Quantitative PCR is often associated with issues in reproducibility and excessive workload, such as the need to create multiple technical replicas to ensure statistical robustness. Pairwise Efficiency provides a significant increase in the precision of estimation of efficiency and gene expression ratio without increasing the workload. According to our analysis, 2–5 individual fluorescence readings from each amplification curve can be taken directly for the estimation of reaction efficiency. Six amplification curves from only six wells (which is three times less than required for calibration curve analysis) can provide 50–200 individual pairwise E determinations, enabling much more extensive statistics. This significantly reduces the workload necessary for achieving high precision.

Another advantage of Pairwise Efficiency is that it relies on actual fluorescence readings rather than implied data. It has been previously pointed out that the estimation of efficiency by the means of a calibration curve, as required by MIQE guidelines, is based not on existing, but rather on implied data: “the data from a tube is discontinuous; fluorescence is measured at the end of each cycle, and there is no such thing as a fluorescence after a fractional number of cycles as implied by the continuous functions [that the classical Cq approach involves]” [25]. We agree with this point of view. One of the advantages of Pairwise Efficiency is that it is based on the analysis of actual fluorescence readings produced after each cycle, and does not rely on fractional cycles.

Finally, Pairwise Efficiency can be distinguished from other approaches because it allows the elimination of outlier values during the process of calculating the efficiency, and not at the end, as is the case in other methods. For example, the MIQE guidelines require that the efficiency be estimated from the slope of the calibration curve, and considers efficiency value E to be the indicator of the robustness of the assay. In cases in which the E value exceeds the theoretical maximum of 100%, it is taken to be the result of reaction inhibition in one of the wells, generally meaning that the entire assay needs to be repeated or redesigned [5]. In contrast, because Pairwise Efficiency provides more than 150 individual E determinations for a single replica of the calibration curve, it makes it possible to apply both the distribution analyzes for normality and the appropriate statistical instruments for eliminating outliers. In this respect, Pairwise Efficiency strongly differs from the classical methods where one or two “outlier” wells would often require the user to re-perform the entire experiment. In Pairwise Efficiency, not only can we obtain more than 150 data points from a single dilution set (six wells), but replication of the calibration curve three times could potentially increase this number up to 2556 (72 fluorescence readings, all in cross-pairwise relationships). This allows the use of powerful statistical instruments, and represents a marked advantage over other methods.

Overall, our new method, Pairwise Efficiency, allows a nearly two-fold increase in the precision of efficiency estimation and a 2.3-fold increase in the precision of the gene ratio estimation (Table 2 and Results). Further refinements to our approach, such as testing the application of different fitting and regression methods, can be explored. We hope that Pairwise Efficiency will become a useful tool for the community and that our efforts will stimulate further investigations in improving the reliability of qPCR determinations.

## Conclusion

To summarize, we have developed a new combinatorics-based method, Pairwise Efficiency, for data analysis in RT-qPCR procedure. Pairwise Efficiency takes advantage of the availability of fluorescence data, introduces a new formula for efficiency calculation by pairwise combinations, and allows to create an array of E values to enable statistical analysis. As a result, the application of Pairwise Efficiency nearly doubles the precision in qPCR efficiency determinations compared to standard calibration curve method, when applied to serial dilutions. Our work makes an example of what can be achieved in RT-qPCR field through combinatorics, and suggests that further applications of combinatorial treatment of data may benefit the qPCR field in general.

## Additional files

Additional file 1: **Table S1.** The ‘first outliers’ calculated by the formula from Tichopad et.al, 2003. **Table S2.** Calculated first derivative (FD) values for the first calibration curve replica (wells A1-A6). **Table S3.** Efficiency values obtained by the standard curve method for all 16 replicas of a dilution set. **Table S4.** The efficiency values calculated with the formula for the mean efficiency (4) with varying F0. **Table S5.** Standard deviations, maximal and minimal efficiency (E) values and their difference, as well as average efficiency for differently set boundaries. **Table S6.** The results of Chi-square test on all 16 identical six-sets from Dataset 1. **Table S7.** Outlier elimination process. **Table S8.** Baseline subtracted fluorescence data analysis by the Pairwise Efficiency method. **Table S9.** Baseline subtracted curve fit fluorescence data analysis by the Pairwise Efficiency method. **Table S10.** Pairwise Efficiency method applied to 10-fold dilution series. **Figure S1.** Agarose gel of the PCR product and melting curve analysis. **Figure S2.** Pipetting layout of the plate. **Figure S3.** The first derivative (FD) values and the corresponding fluorescence (RFU) values for 16 replicas of a 6-step serial dilution set taken from Dataset 1. **Figure S4.** Schematic representation of Monte Carlo simulation for assessment of precision. **Figure S5.** Determination of the most suitable RFU boundaries for a 6-step dilution series. **Figure S6.** Noise values and distribution in the beginning cycles of amplification. **Figure S7.** A graphical representation of the distribution of pairwise E values for the wells H7-H12 compared to normal distribution. (PDF 729 kb)

Additional file 2: Dataset 1. (XLS 67 kb)

Additional file 3: Dataset 2. (XLS 19 kb)

## Data Availability

All data generated or analyzed during this study are included in this published article [and its supplementary information files].

## Abbreviations

DNA: Deoxyribonucleic acid
FDM: First derivative maximum
MIQE: Minimum information for publication of quantitative real-time PCR experiments
qPCR: quantitative polymerase chain reaction
RFU: Relative fluorescence units
RNA: Ribonucleic acid
SD: Standard deviation
