# Supplementary material for: Pairwise efficiency: a new mathematical approach to qPCR data analysis increases the precision of the calibration curve assay
Source: BMC Bioinformatics. 2019 May 30;20:295. doi: 10.1186/s12859-019-2911-5 (PMC6543629; doi:10.1186/s12859-019-2911-5)
Supplement: Supplementary file 1 — Table S1. The ‘first outliers’ calculated by the formula from Tichopad et.al, 2003. Table S2. Calculated first derivative (FD) values for the first calibration curve replica (wells A1-A6). Table S3. Efficiency values obtained by the standard curve method for all 16 replicas of a dilution set. Table S4. The efficiency values calculated with the formula for the mean efficiency (4) with varying F0. Table S5. Standard deviations, maximal and minimal efficiency (E) values and their difference, as well as average efficiency for differently set boundaries. Table S6. The results of Chi-square test on all 16 identical six-sets from Dataset 1. Table S7. Outlier elimination process. Table S8. Baseline subtracted fluorescence data analysis by the Pairwise Efficiency method. Table S9. Baseline subtracted curve fit fluorescence data analysis by the Pairwise Efficiency method. Table S10. Pairwise Efficiency method applied to 10-fold dilution series. Figure S1. Agarose gel of the PCR product and melting curve analysis. Figure S2. Pipetting layout of the plate. Figure S3. The first derivative (FD) values and the corresponding fluorescence (RFU) values for 16 replicas of a 6-step serial dilution set taken from Dataset 1. Figure S4. Schematic representation of Monte Carlo simulation for assessment of precision. Figure S5. Determination of the most suitable RFU boundaries for a 6-step dilution series. Figure S6. Noise values and distribution in the beginning cycles of amplification. Figure S7. A graphical representation of the distribution of pairwise E values for the wells H7-H12 compared to normal distribution. (PDF 729 kb) [file 12859_2019_2911_MOESM1_ESM.pdf]

Pairwise Efficiency: A new mathematical approach to qPCR data analysis increases the precision of the calibration curve assay

Supporting Information

Yulia Panina[1,2] , Arno Germond[1] , Brit G. David[1], Tomonobu M. Watanabe*[1,2]

Laboratory for Comprehensive Bioimaging, RIKEN Center for Biosystems Dynamics Research (BDR)

[1] RIKEN Center for Biosystems Dynamics Research (BDR), 6-2-3 Furuedai, Suita, Osaka 565-0874, Japan

[2] Graduate School of Frontier Biosciences, Osaka University, 1-3 Yamadaoka, Suita, Osaka 565-0871, Japan

*Corresponding author: Tomonobu M. Watanabe, RIKEN Center for Biosystems Dynamics Research (BDR), 6-2-3 Furuedai, Suita, Osaka 565-0874, Japan, Tel: +81-6-6155-0111

Email: tomowatanabe@riken.jp

Contents

Tables

- **Table S1**
- **Table S2**
- **Table S3**
- **Table S4**
- **Table S5**
- **Table S6**
- **Table S7**
- **Table S8**
- **Table S9**
- **Table S10**

Figures

- **Figure S1**
- **Figure S2**
- **Figure S3**
- **Figure S4**
- **Figure S5**

Tables

Table S1. The 'first outliers' calculated by the formula from Tichopad et.al, 2003. The 'first outlier' values calculated for the wells A1 through A6 are denoted in red. The relatively constant values preceding them are denoted in blue. The fluorescence values from Dataset 1 for these wells in the corresponding cycles were A1=14.09 RFU, A2=16.7 RFU, A3=21.28 RFU, A4=27.19 RFU, A5=37.11 RFU, A6=28.2 RFU. Hence, the minimal fluorescence value was RFU=14.09, and the maximal fluorescence value was RFU=37.11. Thus, the tentative lower boundary of the exponential region can be set at approximately 10-40 RFU, depending on the actual curve.

Cycle	A1	A2	A3	A4	A5	A6
3	0.4	0.94	-0.09	1.3	0.31	0.33
4	0.13	0.27	0.21	0.53	0.08	0.77
5	0.1	0.17	0.27	0.24	0.33	0.43
6	-0.01	0.08	0.27	0.26	0.22	0.25
7	-0.05	0.02	0.17	0.23	0.14	0.12
8	0.01	-0.01	0.14	0.16	0.08	0.09
9	0.02	-0.02	0.1	0.12	0.08	0.09
10	0.03	0	0.04	0.1	0.04	0.06
11	0.02	0	0.04	0.08	0.01	0.06
12	0.02	0	0.04	0.07	0.02	0.04
13	**0.05**	0.01	0.04	0.05	0	0.02
14	0.08	**0.03**	0.05	0.04	0.01	0.01
15	0.13	0.05	0.05	0.05	0.01	0.01
16	0.2	0.09	**0.07**	0.05	0.01	0.01
17	0.3	0.15	0.09	0.05	0.02	0.01
18	0.41	0.23	0.13	**0.07**	0.03	0.01
19	0.51	0.33	0.2	0.1	**0.05**	0.02
20	0.6	0.42	0.28	0.14	0.08	**0.03**
21	0.67	0.51	0.36	0.2	0.13	0.06
22	0.71	0.57	0.43	0.27	0.19	0.1
23	0.72	0.61	0.49	0.33	0.25	0.15
24	0.72	0.64	0.53	0.39	0.32	0.2

Table S2. Calculated first derivative (FD) values for the first calibration curve replica (wells A1-A6). Since the wells contain progressively diluted DNA samples, the first derivative maximum (FDM), denoted in red, is reached later and later with each dilution. In these wells, the FDM is reached at cycle 19 for the most concentrated sample, and at cycle 24 for the least concentrated sample.

Cycle	A1	A2	A3	A4	A5	A6
1	-2.46	-3.97	-0.26	-6.41	-2.14	-2.14
2	4.02	7.48	-6.55	3.11	0.59	-3.22
3	-1.67	-0.26	2.02	1.83	-0.36	1.74
4	-0.68	-2.34	1.72	-3.97	-2.35	9.39
5	1.37	2.99	1.78	-2.43	9.6	-5
6	-4.23	-1.37	2.29	5.78	-3.91	-0.34
7	-1.72	-1.23	-3.86	0.45	-0.91	-3.27
8	6.85	-0.89	1.82	-3.29	-1.42	3.01
9	-1.78	1.15	-2.37	-0.13	4.18	3.46
10	2.39	4.27	-6.54	0.15	-5.3	-3.97
11	-4.43	-3.38	7.11	0.11	-4.12	2.99
12	4.69	3.01	-0.15	-0.32	7.77	-4.24
13	10.33	4.14	2.93	-4.48	-5.77	-3.66
14	11.3	9.19	2.28	3.96	7.66	3.35
15	27.09	9.01	4.46	4.29	-4.58	1.09
16	39.84	23.95	10.46	1.24	2.55	1.34
17	67.03	39.86	18.25	5.11	8.85	-2.18
18	73.28	62.22	32.37	17.16	9.54	6.54
19	**76.28**	72.37	62.27	30.88	16.38	10.68
20	66.74	**75.45**	70.92	45.67	27.59	11.62
21	51.29	68.55	**73.46**	64.24	56.89	31.75
22	38.4	51.44	64.14	**66.11**	67.78	42.44
23	28.23	40.81	50.27	60.92	74.28	67.03
24	26.17	35.28	41.62	51.14	**75.52**	**74**
25	17.48	27.78	34.69	38.16	60.34	71.95
26	14.99	22.87	29	32.22	49	62.23
27	7.74	14.81	21.39	25.76	38.59	44.45
28	9.35	14.56	17.09	20.67	32.39	38.6
29	8.53	9.73	12.1	15.79	26.18	32.18

30	6.43	9.21	8.96	11.63	19.11	28.05
31	4.22	5.61	8.37	9.99	16.28	20.18
32	3.23	4.6	8.04	8.16	14.03	14.97

Table S3. Efficiency values obtained by the standard curve method for all 16 replicas of a dilution set. The efficiency values E calculated by the classical calibration curve method are shown for the corresponding wells on the 96-well plate (for pipetting layout see Fig. S2). The Cq data for E calculation was taken from Dataset 2.

	wells 1-6	wells 7-12
**A**	0.801	0.794
**B**	0.838	0.823
**C**	0.797	0.814
**D**	0.882	0.770
**E**	0.778	0.795
**F**	0.763	0.779
**G**	0.808	0.726
**H**	0.839	0.776

Table S4. The efficiency values calculated with the formula for the mean efficiency (4) with varying F0. The values corresponding to the region with relatively constant efficiency (SD=0.01) are denoted in red.

Cycle	F0=0.007	F0=0.0035	F0=0.00175	F0=0.000875	F0=0.0004375	F0=0.0002188
0	0	0	0	0	0	0
1	0	0	0	0	0	0
2	0	0	0	0	0	0
3	0	0	0	0	0	0
4	0	0	0	0	0	0
5	0	0	0	0	0	0
6	0	0	0	0	0	0
7	0	0	0	0	0	0
8	0	0	0	0	0	0
9	0	0	0	0	0	0
10	0	0	0	0	0	0
11	0	0	0	0	0	0

12	0	0	0	0	0	0
13	0.8	0	0	0	0	0
14	0.8	0.83	0	0	0	0
15	0.81	0.81	0.79	0	0	0
16	0.81	0.82	0.8	0	0	0
17	0.8	0.82	0.8	0.73	0.82	0
18	0.78	0.81	0.8	0.78	0.82	0
19	0.76	0.79	0.81	0.79	0.82	0.81
20	0.72	0.76	0.79	0.79	0.81	0.8
21	0.69	0.73	0.77	0.78	0.82	0.82
22	0.66	0.7	0.74	0.76	0.8	0.81
23	0.62	0.67	0.71	0.74	0.78	0.8
24	0.6	0.64	0.68	0.71	0.76	0.79
25	0.57	0.61	0.65	0.68	0.73	0.76
26	0.54	0.58	0.62	0.65	0.7	0.74
27	0.52	0.56	0.59	0.63	0.67	0.71
28	0.5	0.54	0.57	0.6	0.65	0.68
29	0.48	0.51	0.55	0.58	0.62	0.66
30	0.46	0.49	0.53	0.55	0.6	0.63
31	0.44	0.48	0.51	0.53	0.58	0.61
32	0.43	0.46	0.49	0.51	0.55	0.59

Table S5. Standard deviations, maximal and minimal efficiency (E) values and their difference, as well as average efficiency for differently set boundaries are shown. The minimal standard deviation (SD written in bold) is derived when setting the lower boundary at 40 RFU, and the upper boundary at 120 RFU , which falls within the exponential region of the curve. The lowest difference between the maximal E value and the minimal E value (Max-Min difference, in bold) is also observed with the same boundaries (40 - 120 RFU). Note that the average efficiency value tends to decline when the upper boundary is increased in the curve (150, 180, 210, 240) which agrees well with the notion of progressively declining efficiency with the gradual reaction saturation at later cycles. Overall, this result shows that the optimal region for mean efficiency calculation lies within the exponential region of the curve, and that the standard deviation will rise if fluorescence readings from later cycles are included in the calculations.

**Boundaries**	**SD**	**Max E**	**Min E**	**Max-Min difference**	**Average E**

20-150	0.0124	0.8346	0.7779	0.0567	0.8012
30-150	0.0125	0.8339	0.7790	0.0549	0.8039
40-150	0.0124	0.8311	0.7806	0.0505	0.8005
50-150	0.0132	0.8404	0.7750	0.0655	0.8028
60-150	0.0145	0.8485	0.7776	0.0709	0.8044
40-120	**0.0113**	0.8234	0.7798	**0.0436**	0.8012
40-150	0.0124	0.8311	0.7806	0.0505	0.8005
40-180	0.0116	0.8191	0.7675	0.0516	0.7894
40-210	0.0137	0.8274	0.7690	0.0583	0.7926
40-240	0.0165	0.8296	0.7568	0.0728	0.7852

Table S6. The results of Chi-square test on all 16 identical six-sets from Dataset 1. Chi-square test was performed on the groups of pairwise E measurements for each of the six-sets, as indicated. The number of data points (after the removal of statistically unreliable values whose frequency was less than 5, as per Chi-square criteria), the mathematical expectation of each group, standard deviation (SD) of each group, the chi-square value obtained for each group, the degrees of freedom for each group, and the expected Chi value based on the degrees of freedom, are shown. According to the Chi-square test principles, if the Chi-square value exceeds the expected Chi value, the distribution significantly deviates from normal, and parametric statistical instruments, such as quartile ranges or sigma, cannot be applied. Since all 16 six-sets were identical, and the number of non-normally distributed groups was significantly higher (12 out of 16), the analysis of outliers for these data should be performed using non-parametric tools.

**Wells**	**A1-A6**	**A7-A12**	**B1-B6**	**B7-B12**	**C1-C6**	**C7-C12**	**D1-D6**	**D7-D12**
N of data points	218	208	215	233	228	211	205	204
Math. expectation	0.836	0.804	0.822	0.817	0.819	0.821	0.880	0.820
SD	0.096	0.112	0.083	0.085	0.053	0.079	0.101	0.071
Chi-square value	33.727	21.701	12.491	14.503	11.556	15.515	10.004	22.780
Degrees of freedom	9	10	9	8	5	7	9	7
Expected chi-square	16.919	18.307	16.919	15.507	11.07	14.067	16.919	14.067
Does it fit?	No	No	Yes	No	No	No	Yes	No
**Wells**	**E1-E6**	**E7-E12**	**F1-F6**	**F7-F12**	**G1-G6**	**G7-G12**	**H1-H6**	**H7-H12**
N of data points	207	199	247	224	209	199	183	227
Math. expectation	0.812	0.807	0.796	0.804	0.830	0.783	0.840	0.798

SD	0.054	0.079	0.060	0.083	0.062	0.059	0.067	0.080
Chi-square value	7.049	29.687	13.195	5.821	20.549	33.988	19.465	36.350
Degrees of freedom	5	7	6	8	6	6	6	8
Expected chi-square	11.07	14.067	12.592	16.919	12.592	12.592	12.592	16.919
Does it fit?	Yes	No	No	Yes	No	No	No	No

Table S7. Outlier elimination process. The boundaries were set to 20-180. Red values have frequencies less than 5, and denote the boundaries from where outliers were eliminated. Thus, for example, all E measurements lower than 0.6 (60%) were considered outliers for wells A1-A6, and all E measurements greater than 1.15 (115%) were also excluded from the mean E calculation for these wells.

E scale	A1-A6	B1-B6	C1-C6	D1-D6
0	0	0	0	0
0.05	0	0	0	0
0.1	0	0	0	0
0.15	0	0	0	0
0.2	0	0	0	0
0.25	1	1	0	0
0.3	0	0	0	0
0.35	0	0	0	0
0.4	2	0	0	0
0.45	0	1	0	0
0.5	1	3	0	2
0.55	4	1	2	1
0.6	3	8	3	0
0.65	9	8	2	6
0.7	14	22	11	8
0.75	34	46	28	13
0.8	42	55	85	33
0.85	57	40	80	39
0.9	30	27	17	41
0.95	15	14	7	32
1	6	6	3	16
1.05	5	5	1	9
1.1	6	3	0	8

1.15	2	0	1	3
1.2	1	0	0	3
1.25	1	1	0	2
1.3	0	2	0	0
1.35	2	0	0	1
1.4	0	1	0	0
1.45	1	0	0	0
1.5	0	0	0	1
1.55	0	0	0	1
1.6	0	0	0	0
1.65	0	0	0	0
1.7	0	0	0	0
1.75	1	0	0	0
1.8	0	0	0	0
1.85	0	0	0	0
1.9	0	0	0	1
1.95	0	0	0	0
2	0	0	0	0
total N of data points	237	244	240	220
N after outlier removal	218	231	228	205

Table S8. Baseline subtracted fluorescence data analysis by the Pairwise Efficiency method. The "Baseline Subtracted" settings were chosen on the Biorad CFX96 qPCR machine. The boundaries were set to 20-180. This baseline subtraction method produces more fluorescence data points for analysis than the "Baseline Subtracted Curve Fit" (see Table S10).

Well	Efficiency (%)	F0	STD E	total data points	points after outlier removal
A1-A6	0.8009	0.0050	0.2512	258	241
A7-A12	0.7685	0.0048	0.2522	258	233
B1-B6	0.7753	0.0052	0.2434	244	229
B7-B12	0.7834	0.0052	0.2414	241	232
C1-C6	0.7828	0.0055	0.2342	240	233
C7-C12	0.7850	0.0052	0.2421	240	227
D1-D6	0.8369	0.0041	0.2721	237	223

D7-D12	0.7795	0.0040	0.2446	223	202
E1-E6	0.7756	0.0051	0.2324	240	230
E7-E12	0.7859	0.0051	0.2498	217	205
F1-F6	0.7706	0.0050	0.2308	259	239
F7-F12	0.7746	0.0050	0.2426	241	223
G1-G6	0.7964	0.0047	0.2469	223	207
G7-G12	0.7519	0.0046	0.2316	206	200
H1-H6	0.8208	0.0034	0.2629	231	206
H7-H12	0.7688	0.0036	0.2374	240	228

Table S9. Baseline subtracted curve fit fluorescence data analysis by the Pairwise Efficiency method. The "Baseline Subtracted Curve Fit" settings were chosen on the Biorad CFX96 qPCR machine. The boundaries were set to 20-180. Pairwise Efficiency method is applicable to both types of baseline-treated data and gives similar efficiency estimations because the total number of data points do not significantly differ. However, since a little fewer fluorescence data points are available for analysis due to the application of curve fitting, the resulting E will slightly differ.

Well	Efficiency (%)	F0	STD E	total data points	points after outlier removal
A1-A6	0.8105	0.0037	0.2582	237	218
A7-A12	0.7805	0.0050	0.2590	237	208
B1-B6	0.7829	0.0049	0.2447	244	231
B7-B12	0.7930	0.0044	0.2433	241	233
C1-C6	0.7933	0.0047	0.2370	240	228
C7-C12	0.7935	0.0045	0.2462	222	211
D1-D6	0.8537	0.0025	0.2734	220	205
D7-D12	0.7949	0.0044	0.2486	223	204
E1-E6	0.7866	0.0050	0.2402	220	207
E7-E12	0.7831	0.0051	0.2491	217	199
F1-F6	0.7702	0.0053	0.2287	259	247
F7-F12	0.7775	0.0050	0.2418	241	224
G1-G6	0.8044	0.0036	0.2466	223	209
G7-G12	0.7574	0.0060	0.2328	206	199
H1-H6	0.8151	0.0030	0.2597	198	183
H7-H12	0.7736	0.0049	0.2389	240	227

Table S10. Pairwise Efficiency method applied to 10-fold dilution series. The Efficiency value was calculated for each of the six-well set by the Pairwise Efficiency method, based on the number of fluorescence data points indicated in the column “total data points”. Efficiency C values, on the other hand, were calculated by the classical calibration curve approach. Negative Efficiency C values indicate the failure of the method to calculate meaningful efficiency due to errors in some of the wells (see the extremely small numbers of data points for these sets). While the calibration curve gave several negative values, indicating failure, the Pairwise Efficiency method could still give a satisfactory result, even when the number of data points was small.

Well	Efficiency (%)	Efficiency C (%)	F0	Ratio	total data points
A1-A6	0.899	0.895	0.00127	1.00	91
A7-A12	0.937	0.956	0.00126	0.99	105
B1-B6	0.902	0.906	0.00081	1.00	120
B7-B12	0.991	-0.819	0.00094	1.16	45
C1-C6	0.902	-0.854	0.00160	1.00	78
C7-C12	0.948	-0.823	0.00154	0.96	78
D1-D6	0.897	0.920	0.00108	1.00	136
D7-D12	0.922	0.952	0.00123	1.14	119
E1-E6	0.941	1.012	0.00156	1.23	119
E7-E12	0.877	0.883	0.00118	0.93	105
F1-F6	0.955	0.977	0.00095	1.18	120
F7-F12	0.904	0.935	0.00082	1.02	104
G1-G6	0.861	0.861	0.00153	0.96	120
G7-G12	0.847	0.865	0.00154	0.96	78
H1-H6	0.901	0.935	0.00112	1.03	120
H7-H12	0.934	0.971	0.00130	1.21	120

Figures

(a)

(b)

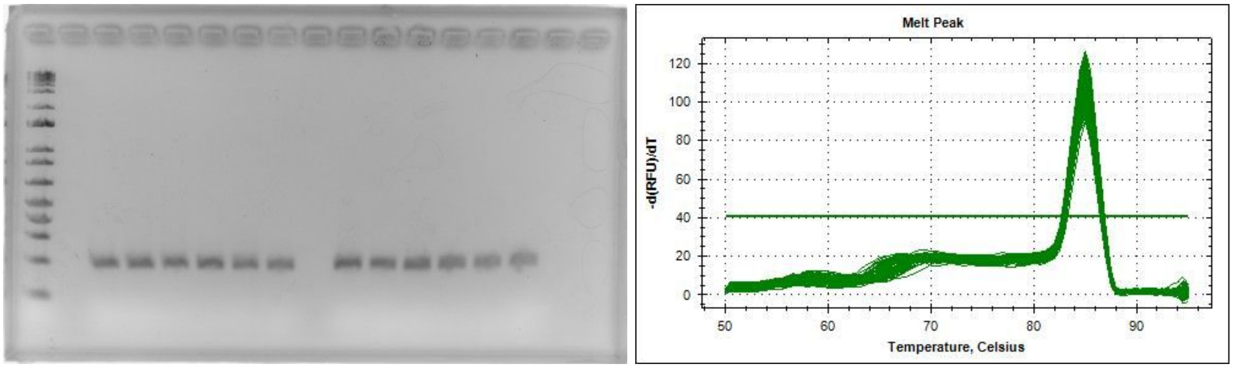

Figure S1. **Agarose gel of the PCR product and melting curve analysis.** **(a)** Agarose gel confirms the amplification of the expected product, showing a band at 194 bp. **(b)** Melting curve analysis shows no primer dimers and a single sharp peak, as expected.

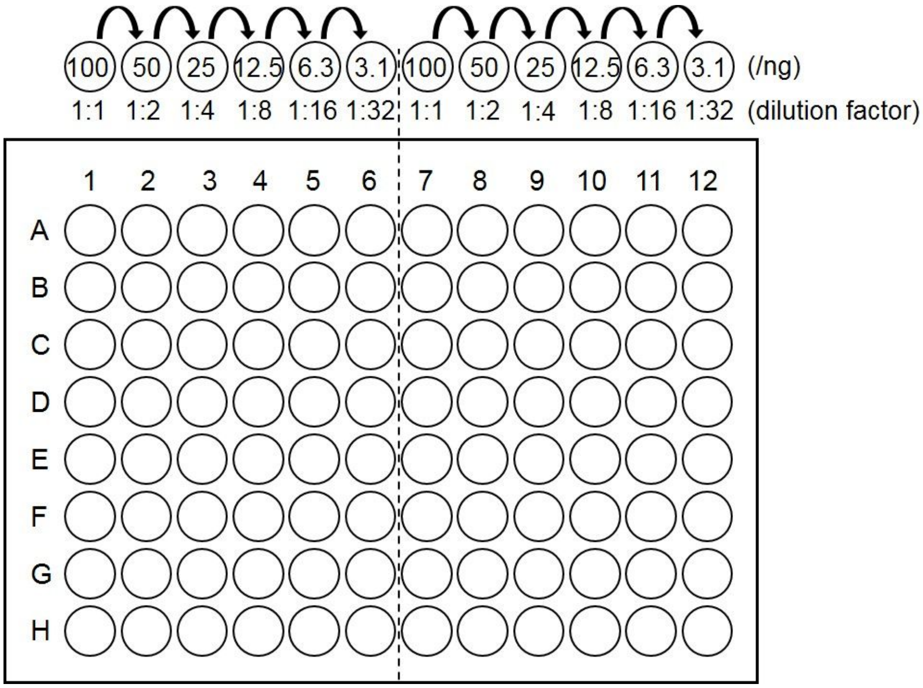

Figure S2. **Pipetting layout of the plate.** A dilution set consisting of 6 serial two-fold dilutions was replicated 16 times. The dilution factor (D) used in the formula in the main text refers to these dilution steps, and can assume the following values (in case of 6-step dilution set): 1, 2, 4, 8, 16 and 32. For demonstration purposes, a two-fold serial dilution instead of the usual 10-fold serial dilution was used in this experiment, as it provides mathematical clarity (see Results for further explanation).

(a)

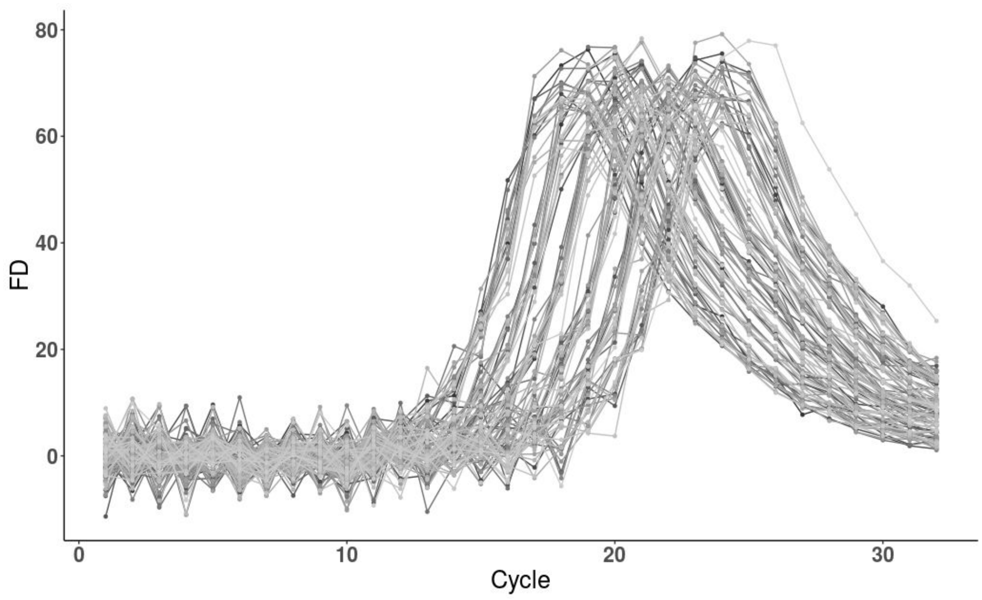

(b)

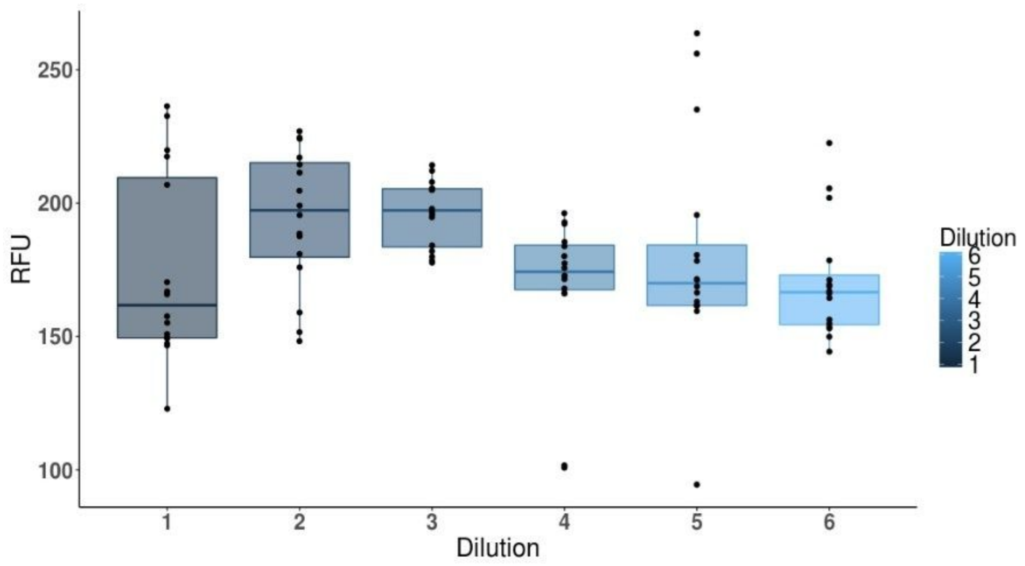

Figure S3. The first derivative (FD) values and the corresponding fluorescence (RFU) values for 16 replicas of a 6-step serial dilution set taken from Dataset 1. (a) First derivative values of 96 amplification curves were plotted against the cycle at which they were obtained. As the dilution factor increases, the FD values are delayed and come at later cycles. The maximum of the first derivative (FDM) for the most concentrated sample corresponds to cycle 18, while for the least concentrated sample this occurs at cycle 25. **(b)** The obtained FDM values were plotted against corresponding fluorescence units at the same cycle. Horizontal lines across the boxes denote the mean. Black dots indicate individual values, and the colors correspond to dilution (most concentrated sample - dark blue, least concentrated sample - light blue). The majority of FDM values roughly correspond to a RFU of 150-230.

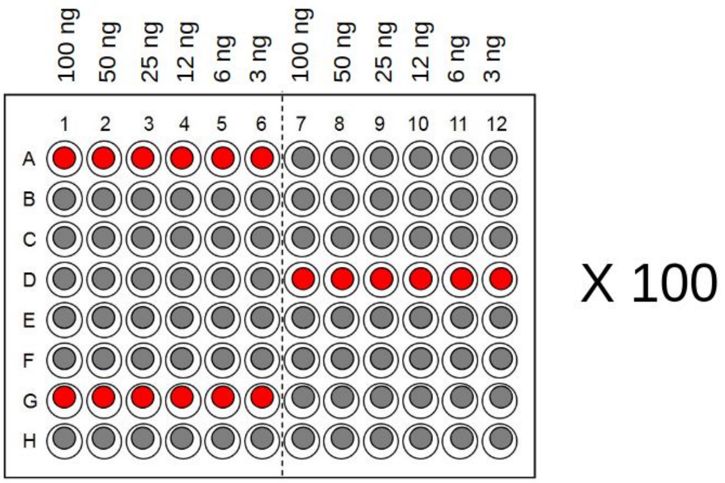

Figure S4. Schematic representation of Monte Carlo simulation for assessment of precision. All 96 wells contain samples of different concentration (written above the plate). Technically, this constitutes 16 sets of six-step dilution series, 8 on the left half of the plate, and 8 on the right half of the plate. These 16 sets are identical and represent the general population on which Monte Carlo simulation is based. For each assessment of SD of a given boundary set (for example, for boundaries 10 RFU-180 RFU), 100 pseudo-random measurements are performed using Pairwise Efficiency method. Each pseudo-random measurement consists of averaged 3-set of randomly chosen dilution series (represented by red circles in the figure). The SDs of different boundaries are then compared to each other.

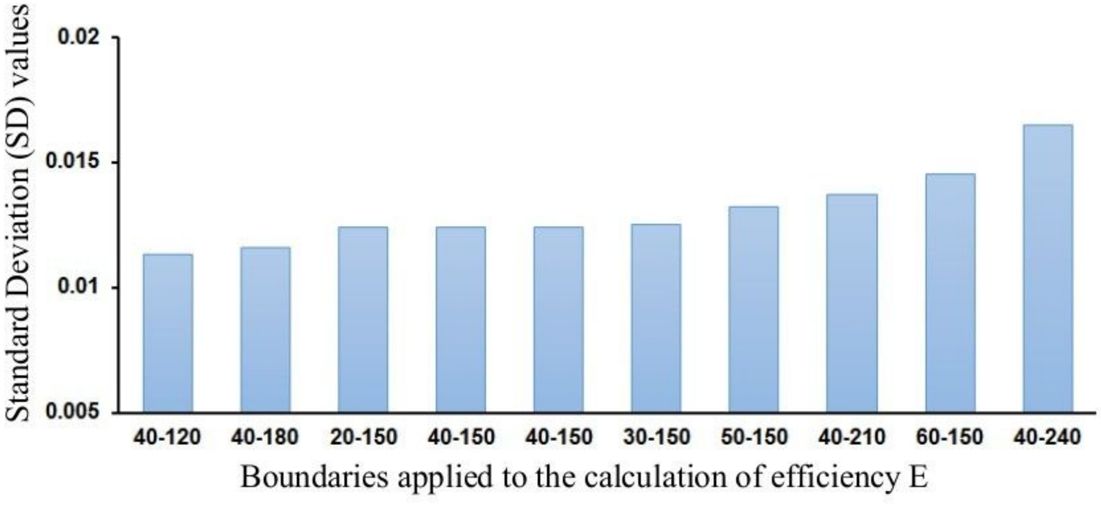

Figure S5. **Determination of the most suitable RFU boundaries for a 6-step dilution series.** Standard deviations (SD) of the efficiency values calculated by Monte Carlo approach using different regions of amplification curves. Each time different portions of the amplification curves (defined by different boundaries for RFU) were included in the calculations. The lower boundary

varied between 20 RFU and 80 RFU, while the upper boundary varied from 120 RFU to 240 RFU. The lowest SD was obtained when applying the following boundaries: lower at 40 RFU and upper at 120 RFU. The SD tended to rise when boundaries were raised.

(a)

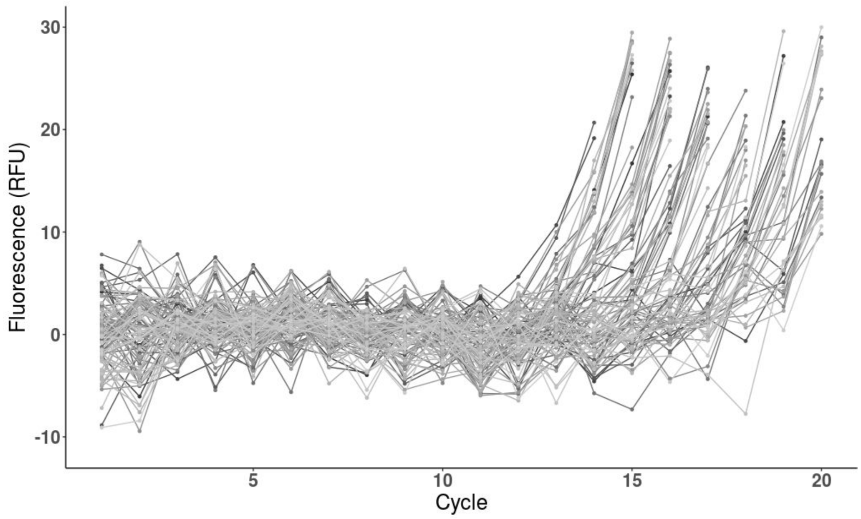

(b)

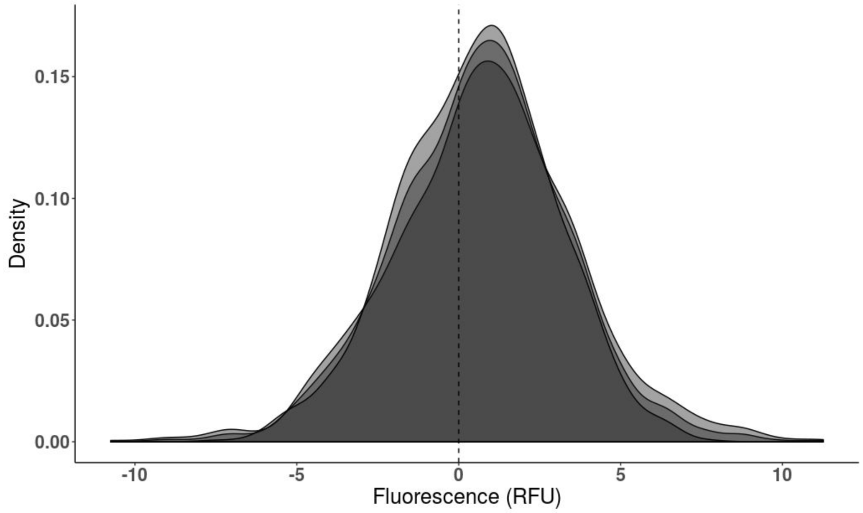

Figure S6. **Noise values and distribution in the beginning cycles of amplification. (a)** Amplification curves showing the beginning cycles for 96 qPCR reactions from Dataset 1. The noise is distributed close to zero and the noise phase appears to continue up to cycle 13. **(b)** Distribution of the noise across 2880 qPCR reactions taken from Dataset 1. The graph shows three groups of noise values: cycles 1-5, cycles 5-10 and cycles 1-10. All groups have nearly normal distribution with a non-zero mean, not shifting with increasing cycles, and the maximal data values reach approximately 10 fluorescence units.

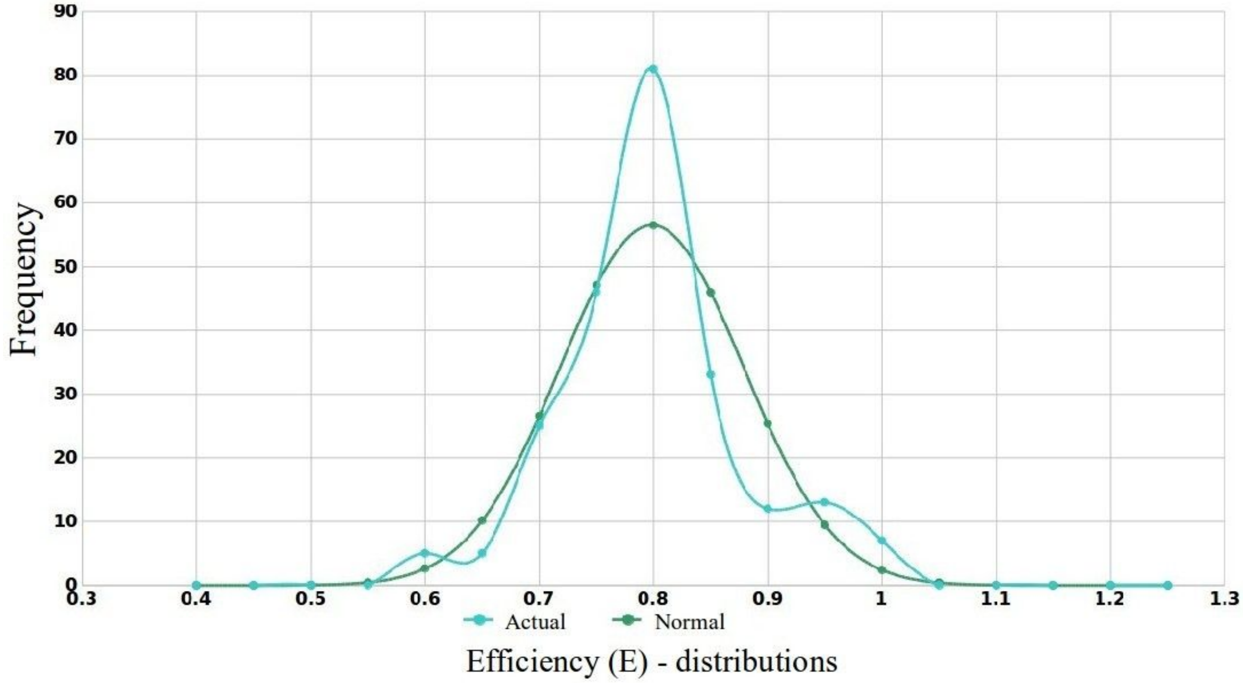

Figure S7. **A graphical representation of the distribution of pairwise E values for the wells H7-H12 compared to normal distribution.** The distribution of pairwise E values is leptokurtic (has a sharp peak), indicating that the values are closer to mathematical expectation, and that the precision is higher than would be expected in the case of normal distribution. In addition, skewness is present in this distribution as compared to normal distribution, indicating significant deviation from normality.
